# Vector abundance and associated abiotic factors that influence the distribution of ticks in six provinces of South Africa

**DOI:** 10.14202/vetworld.2024.1765-1777

**Published:** 2024-08-13

**Authors:** Tsireledzo G. Makwarela, Appolinaire Djikeng, Tracy M. Masebe, Nyangiwe Nkululeko, Lucky T. Nesengani, Ntanganedzeni O. Mapholi

**Affiliations:** 1 Department of Life and Consumer Sciences, College of Agriculture and Environmental Sciences, University of South Africa, Private Bag X6, Florida 1710, South Africa; 2 Department of Agriculture and Animal Health, College of Agriculture and Environmental Sciences, University of South Africa, Private Bag X6, Florida 1710, South Africa; 3 Centre for Tropical Livestock Genetics and Health, Royal (Dick) School of Veterinary Studies, University of Edinburgh, Edinburgh EH8 9YL, UK

**Keywords:** abiotic factors, cattle infestations, climatic conditions, South Africa, ticks, vector abundance

## Abstract

**Background and Aim:**

Climatic conditions significantly impact the life stages and distribution patterns of ticks and tick-borne diseases. South Africa’s central plateau and various biomes offer a distinct landscape for studying the geography’s effects. The study estimated tick species prevalence and the influential factors on their survival.

**Materials and Methods:**

Ticks were gathered from communal cattle in South African provinces including Limpopo (LP), Gauteng (GP), Mpumalanga (MP), KwaZulu-Natal (KZN), the Eastern Cape (EC), and the Free State (FS), from September 2020 to November 2022. Using data from South African weathercasts, the annual climate was assessed.

**Results:**

A total of 3,409 ticks were collected, with the highest infestation observed in KZN (45%), followed by LP (26%), EC (19%), GP (5%), MP (2%), and the FS (2%). The most prevalent tick species were *Amblyomma hebraeum* (55.1%), *Rhipicephalus evertsi evertsi* (13.9%) and *Rhipicephalus* (*Boophilus*), *and decoloratus* (11.9%). Other species included *R*. (*Boophilus*) *microplus* (10.85%), *Hyalomma marginatum* (4.8%), *Rhipicephalus appendiculatus* (1.4%), *Harpalus rufipes* (0.8%), *Rhipicephalus exophthalmos* (0.2%), *Rhipicephalus glabroscutatus* (0.2%), *Rhipicephalus sanguineus* (0.2%)*, Haemaphysalis silacea* (0.5%), *Ixodes pilosus* (0.1%), and *Rhipicephalus simus* (0.1%). The infestations were most prevalent on farms in Pongola and KZN. The temperature fluctuated between 12°C and 35°C during data gathering, while humidity varied between 40% and 65%.

**Conclusion:**

This study showed that ticks survive optimally in warm temperatures and high humidity conditions. Livestock farms with high tick infestations may be associated with several risk factors. These practices could involve suboptimal grazing, insufficient acaricidal treatment, and detrimental effects resulting from traditional animal husbandry. Future research is needed to longitudinally evaluate the effects of climate change on tick populations, pathogen transmission, hosts, habitats, and human behavior, influencing potential exposure risks.

## Introduction

Given their ability to adapt to various locations, climatic conditions, and host species, ticks are among the most significant arthropod parasites [1, 2]. The distribution and population density of certain tick species are determined by climate, landscape features, and host presence [3]. Ixodid ticks, the most diverse group of ticks in the Ixodida order, can survive in tropical and subtropical regions while transmitting pathogens leading to significant losses in livestock [4]. In South Africa, Ixodid ticks of several genera, including *Amblyomma*, *Dermacentor*, *Haemaphysalis*, *Hyalomma*, *Ixodes*, *Rhipicephalus*, and *Boophilus* subgenus, prefer cattle as hosts [5]. South Africa’s climate is shaped by its eastern and western coasts, which are oceanic, and its interior plateaus. Shifts in endemic tick species, ecological traits, and local host populations along with the emergence of human and animal diseases can be among the far-reaching consequences of tick distribution changes and invasions into new areas [6–8].

Ticks survive and reproduce depending on both biotic and abiotic conditions [9]. The global mean surface temperature is predicted to rise between 0.3°C and 0.7°C from 2016 to 2035 compared to the 1986–2005 period, as per Fan *et al*. [10]. Changes in climate conditions are anticipated to impact the range and presence of ticks as well as the frequency of ticks and tick-borne diseases [11]. The report by Ogden *et al*. [12] shows that ticks’ distribution is affected by climatic conditions, increasing mortality and decreasing productivity. The study also indicated potential indirect impacts on tick infestation, host populations, and tick habitats. The species specificity and temperature changes significantly impact the survival of ticks in diverse environments [13]. Yoder *et al*. [14] highlighted that those terrestrial arthropods, such as ticks, experience diminished moisture retention when exposed to temperatures ranging from 40°C to 45°C. Some species of *Amblyomma hebraeum* suffer water loss in 70% humidity while certain *Rhipicephalus* species thrive with 66% humidity [15]. Moreover, *Rhipicephalus sanguineus* (Latreille) demonstrates resilience to dry air and temperatures ranging from 18°C to 38°C, whereas *Rhipicephalus appendiculatus* stands out as one of the most heat-tolerant species capable of enduring temperatures as high as 40°C, even thriving in environments with low relative humidity [16]. Finally, *Rhipicephalus evertsi evertsi* has specific humidity requirements, 91%–93.5% for fully engorged nymphs and 82%–84.5% for adult ticks [17].

The relationship between tick distribution and climate in South Africa remains unexplored due to insufficiently distributed meteorological stations. To predict and prevent the emergence and re-emergence of tick-borne diseases for public and animal health, as well as the implementation of control measures, it is essential to study tick distribution due to climate change. This study aimed to estimate the prevalence and contributing factors to the tick spread from cattle in the study areas.

## Materials and Methods

### Ethical approval

Ticks were collected from natural grazing communal cattle during the dip period. Animal health technicians from the Department of Agriculture at the target study sites assisted with the removal of ticks while ensuring that the cattle remained unharmed. Ethical approval was obtained from the University of South Africa-College of Agriculture, Engineering, and Science (CAES) Animal Research Ethics Committee (Rec Ref #: 2022/CAES-AREC/036) and the Department of Agriculture, Forestry, and Fisheries (Ref: 12/11/1/1/23 [1466AC]).

### Study period and location

**Figure-1:**
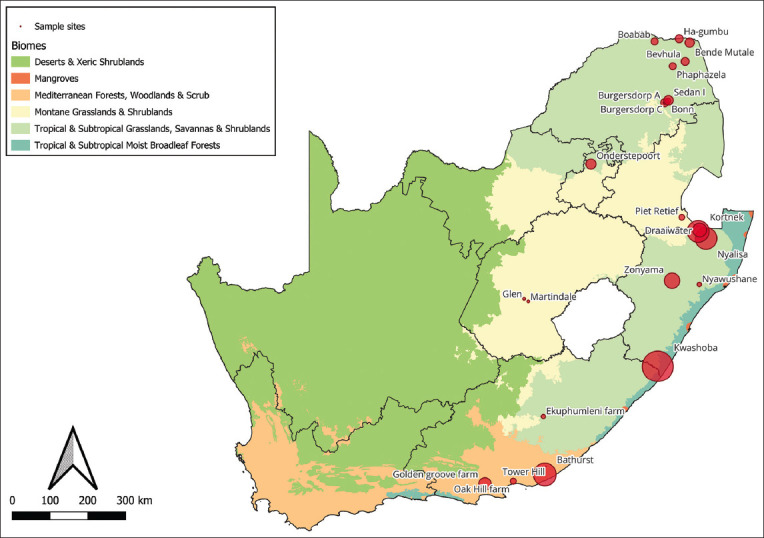
A map of South Africa, highlighting the study sites from which hard ticks were collected. The green letters denote the specific provinces concerned, whereas the red dots signify the locations of the 21 dip tanks from which the collection of ticks occurred: n = 9 Limpopo (LP), n = 1 Gauteng (GP), n = 1 Mpumalanga (MP), n = 4 KwaZulu-Natal (KZN), n = 2 Free State (FS), and n = 4 Eastern Cape (EC), where “n” represents the number of localities per province [Source: The map ourselves using QGIS software, version 3.4.5].

The study was conducted from September to November 2020 and from September to November 2022. Ixodid ticks were gathered from various farms across Limpopo (LP), Mpumalanga (MP), KwaZulu-Natal (KZN), Gauteng (GP), the Free State (FS), and Eastern Cape (EC) in South Africa (Figure-1).

### Experimental cattle

Cattle from six provinces in South Africa were randomly chosen, irrespective of age and gender. Farmers were asked to bring their cattle to the dip tanks for tick collection. Following tick examination and removal, the cattle were dipped for tick control. The entire animal cohort remained healthy throughout the procedure.

### Location, geography, and collection period

The study sites were chosen according to the agrogeographical zones characterized by the dominant tick species. The altitude of these collection sites is between 1000 m and 2100 m above sea level.

South Africa lies between two oceans, the Atlantic and Indian, resulting in diverse climatic zones. Ticks were gathered in autumn 2020 during the months of September, October, and November. The execution of supplementary collections was conducted in the same months of 2022. Ticks belonging to the genera *Amblyomma*, *Hyalomma*, *Ixodes*, *Rhipicephalus*, and subgenus *Boophilus* were gathered for study. Table-1 summarizes the site characteristics.

**Table-1:** Summary of the site characteristics of the six provinces investigated in this study.

Site character-istics	Limpopo				KwaZulu-Natal	Eastern Cape		Mpumalanga	Gauteng	Free State
					
Tzaneen	Mutale	Musina	Thoho-yandou	Pongola	Gqeberha	Bathurst	Mkhondo	Pretoria	Bloem-fontein
District	Mopani	Vhembe	Vhembe	Vhembe	Zululand	Chris-Hani	Sarah-Baartman	Gert Sibande	Tshwane	Motheo
Climate	Sub-tropical	Sub-tropical	Hot semi-arid	Sub-tropical	Sub-tropical	Sub-tropical	Sub-tropical	Tropical	Sub-tropical	Sub-tropical
Latitude Longitude	23.8320° S 30.1358° E	22.5108° S 30.8039° E	22.3529° S 30.0341° E	22.8785° S 30.4818° E	27.3831° S 31.6198° E	33.9608° S 25.6022° E	33.5864° S 26.8851° E	27.0245° S 30.7925° E	25.7479° S 28.2293° E	29.0852° S 26.1596° E
Altitude (m)	719	625,52	542,38	553	234	60	83	1251	1339	1395
Mean annual rainfall (mm)	881	681	372	825,5	806	563	596	954	661	545
Mean annual temp (°C)	19,7	25,65	22,9	20,65	20,2	18,1	18,9	16,1	18,4	17,1

### Description of the study site

South Africa, encompassing over 2,500 km, is bordered by Namibia, Botswana, Zimbabwe, Mozambique, Swaziland, and the Indian and Atlantic oceans. The country lies between longitudes 16°E to 33°E and latitudes 22°S to 35°S. The plateau interior of this landscape ranges from 1,000 to 2,100 m in altitude, with the highest points occurring in the eastern part, and gradually descending toward the west, north, and south-west (McCarthy, 2013).

South Africa has three main soil regions. Soils in the eastern part, roughly east of longitude 25°E, include laterite, unleached subtropical, and podzolic types. These soils formed under summer rainfall and winter dry conditions. The Western and EC’s second notable region, characterized by perpetual precipitation, exhibits grey sand and sandy loam soils. The country is predominantly covered by sandy soil. Rare extreme temperature fluctuations occur throughout much of the temperate zone in the country.

Areas in South Africa mostly have stable weather due to their proximity to the subtropical high-pressure belt with descending air masses. The reasons stated lead to a generally dry climate. More than half of the country, consisting of the semi-arid region, receives between 200 and 600 mm of rainfall annually, while the dry region receives <200 mm/year.

In mountainous regions, temperatures are relatively low, while warmer conditions are typical in the northern and Northeastern areas, western plateaus, and central and southern river valleys. Winters often have cool to freezing night-time temperatures and relatively mild daytime highs between 10°C and 21°C, leading to limited tick populations. Winters along the eastern and southern coastlines are less severe. Temperature levels tend to decrease from east to west.

South Africa boasts a rich variety of wildlife, with over 200 different species of mammals inhabiting the country. This ecosystem is inhabited by different antelope species and larger animals including lions, leopards, elephants, rhinoceroses, hippopotamuses, baboons, and zebras. Mongooses, jackals, and several cat species, including the caracal, make up the smaller inhabitants of the ecosystem. Ticks with multiple host cycles contribute significantly to tick infestation in diverse ecosystems.

### Land use, vegetation, and farming types

The present study revealed intriguing regions and landscapes where the research took place, as highlighted in Table-2. Each area boasts unique environmental and economic characteristics.

**Table-2:** A comprehensive summary of the collection sites involved, the total number of ticks counted, the range of vegetation types in the different areas, the tick control methods employed, and the landscape characteristics. This table offers a clear and organized overview of the different study sites, providing essential contextual information for understanding tick populations, their habitats, and the factors influencing tick infestation in the relevant regions.

Province	Town	Localities	Ticks collected	Grazing system	Vagetation type	Tick-control methods	Landscape
Limpopo	Tzaneen	Bonn	85	Rangeland, grazing	Bushveld	Dip	Moutainious
		Burgersdorp A	110	Rangeland, grazing	Bushveld	Dip	Moutainious
		Burgersdorp C	46	Rangeland, grazing	Bushveld	Dip	Moutainious
		Sedan I	131	Rangeland, grazing	Bushveld	Dip	Moutainious
	Mutale	Ha-gumbu	109	Rangeland, grazing	lowveld and wooldlands	Dip	plain
		Bende Mutale	127	Rangeland, grazing	lowveld and wooldlands	Dip	plain
		Bevhula	109	Rangeland, grazing	lowveld and wooldlands	Dip	plain
	Musina	Boabab	97	Feedlots	Thorn forests and shrubs	Spraying	valley floor
	Thohoyandou	Phaphazela	95	Rangeland, grazing	Bushveld	Spraying	Moutainious
KwaZulu Natal	Pongola	Kwashoba	426	Rangeland, grazing	Sub-escarpment savanna	Dip	Moutainious
		Zonyawa	212	Rangeland, grazing	Sub-escarpment savanna	Dip	Moutainious
		Nyalisa	300	Rangeland, grazing	Sub-escarpment savanna	Dip	Moutainious
		Kortnek	301	Rangeland, grazing	Sub-escarpment savanna	Dip	Moutainious
		Draaiwater	192	Rangeland, grazing	Sub-escarpment savanna	Dip	Moutainious
		Nyawushane	60	Rangeland, grazing	Sub-escarpment savanna	Dip	Moutainious
Eastern Cape	Gqeberha	Tower Hill	11	Pasture, grazing	Savanna	Spraying	Moutainious
		Ekuphumleni farm	57	Pasture, grazing	Albany thickets	Spraying	Moutainious
		Oak Hill farm	79	Pasture, grazing	Savanna	Spraying	Moutainious
		Golden groove farm	172	Pasture, grazing	Savanna	Spraying	Moutainious
	Port alfred	Bathurst	314	Pasture, grazing	Sub-escarpment savanna	Spraying	Moutainious
Mpumalanga	Piet Retief	Piet Retief	79	Rotational grazing	Grassland	Spraying	Undulating hills
Gauteng	Pretoria	Onderstepoort	139	Rotational grazing	Grassland	Spraying	Flat
Free state		Martindale	37	continuous grazing	highveld grassland	Spraying	Flat
		Glen	39	Rotational grazing	highveld grassland	Spraying	Flat

### Pongola

Pongola, famed for its extensive subtropical fruit farms surpassing 50 km^2^, is a peaceful subtropical area. In this area, agriculture flourishes, especially in the cultivation of subtropical fruits. Its favorable climate and rich orchards facilitate substantial economic growth through fruit cultivation. In Pongola, the temperature ranges from 12°C to 33°C during the wet season when conditions are warm and humid.

### Tzaneen

Tzaneen, a subtropical region, boasts diverse vegetation, including native and exotic plant species. The northern Drakensberg’s forest base hosts South Africa’s largest subtropical fruit production area. Many of the tropical forests, once historic, have been turned into plantations for pine, fruit crops, and other agricultural products. Tzaneen’s economy flourishes through fruit farming, vegetable cultivation, livestock rearing, and timber production. Tzaneen experiences an annual temperature swing of 8°C–29°C, accompanied by short, cool winters and extended summers that can be foggy. The varying climate conditions in South Africa contribute to both its rich ecological makeup and diverse agricultural practices.

### Mutale

Mutale lies near the imposing Soutpansberg Mountains. This region borders Kruger National Park, enabling cattle grazing at its edges and overlapping water sources with abundant wildlife. In drought years, some cattle migrate to Zimbabwe for pasture along the river. Mutale encompasses various ecosystems: sandstone cliffs, lush riverine forests, mixed sandvelds, woodlands, and tropical floodplains. This region is marked by low shrubs and thorny trees, a dry climate, and a major mining area. Annually, temperatures range from 11°C to 32°C in this region, characterized by hot and arid conditions.

### Gqeberha

Gqeberha, situated along South Africa’s south-eastern seaboard, includes six of the country’s seven biomes, demonstrating an exceptional ecological diversity encompassing thicket, grassland, Nama-Karoo, fynbos, and forest biomes. The region’s landscape is characterized by east-west-aligned mountain ranges and valleys. In the city’s core, residential, commercial, and retail activities prevail, while small-to-medium-scale farming, including animal husbandry and crop cultivation, characterizes the outskirts. Gqeberha experiences a climate defined by dry, windy conditions, with warm summers averaging 26°C and cool winters averaging 8°C.

### Mkhondo

Mkhondo, situated at the intersection of major roads such as the N2, R543, and R33, is economically sustained by its extensive pine forest plantations. In Mkhondo, the forest industry, particularly the plantations, significantly contributes to the local economy. The Mkhondo climate distinguishes its wet season by warmth and partly cloudy skies, while its dry season is identified by comfortable conditions and mostly clear skies. The temperature usually fluctuates between 4°C and 26°C, with exceptional occurrences below 1°C or above 30°C.

### Pretoria

Pretoria lies as a transitional city between the Southern Highveld plateau and the Northern Bushveld. The Magaliesberg Mountains, with their surrounding fertile valley, characterize Pretoria’s distinctive landscape. The area is characterized by well-established neighborhoods, thriving business districts, and agricultural lands to the south. In the Valley of Magaliesberg Mountains, Pretoria boasts long, warm summers and short, cool winters, with an average temperature ranging from 5°C to 28°C.

### Thohoyandou

Thohoyandou, a significant cultural and economic hub in the LP province of South Africa, serves as the administrative capital of the Vhembe District Municipality. Thohoyandou boasts an unusual climate, characterized by long, hot, humid summers and brief, cool, and dry winters. In Thohoyandou, the annual temperature ranges from 10°C to 29°C, although temperatures occasionally dip below 7°C or climb above 35°C.

### Bloemfontein

Bloemfontein, South Africa’s central location, serves as a critical transportation and economic hub. The city’s unique economic importance results from its location on the Highveld plateau. Bloemfontein, a hub for government activities, commerce, and education, experiences mild summers and short, cool, and dry winters. The city’s temperature ranges from an average of 2°C–30°C, with only occasional instances below 6°C or above 34°C. These fluctuations result in the entire region exhibiting various seasonal weather patterns.

### Bathurst

The Sunshine Coast of South Africa, where Bathurst is situated, is marked by the Kowie River that empties into the Indian Ocean. This quaint town boasts scenic beaches, riverside views, a thriving tourist industry, a prosperous agricultural sector, and a vibrant small business community. As a commercial hub for its vicinity, it caters to residents and tourists with multiple amenities. The summers are short, warm, and humid, while the winters are long, cool, and windy. The temperature in Bathurst fluctuates annually between 12°C and 26°C, with exceptions rarely going below 9°C or above 30°C.

Understanding the complex relationship between environmental factors, tick populations, and livestock is crucial to grasping the diversity of landscapes, ecosystems, and economic activities across the regions studied in this research.

### Climate data collection

For the present study, climate data including temperature, humidity, and rainfall were collected from the reliable online platform, Weatherspark (https://weatherspark.com). After the data were retrieved, patterns and trends were identified through analysis. Graphs were created to effectively illustrate the noted climate fluctuations. Microsoft PowerPoint 2016 was used to effectively display and organize the data visuals.

### Sampling methods

During dip seasons, communal cattle were surveyed for the presence of hard ticks (*Ixodidae*). The cattle under consideration were thoroughly checked from head to tail for evidence of tick infestation. Briefly, following protocols first implemented by Patton *et al*. [18] and Mărcuțan *et al*. [19], adult ticks were carefully removed from the cattle skin using sharp forceps, while ensuring that the external structure of each tick remained intact to retain characteristics constituting the necessary features for morphological identification. The ticks were preserved in vials containing 70% ethanol. During transportation to the University of South Africa’s Florida Science Campus in the GP Province, the cooler box containing the vials was sealed tightly with biohazard tape and kept cool using ice packs. The samples were refrigerated at 4°C.

### Tick counting

At various collection sites, tick species were initially grouped together based on their resemblance. Trained field technicians from the Department of Agriculture carried out the tick collection at the study sites. Two technicians were designated for tick removal while one counted the cattle during the roundup. The researcher in charge overseeing the entire project sorted, counted, and prepared the ticks for further analysis by placing them in vials.

### Statistical analysis

The study employed IBM SPSS Statistics 26.0 software (IBM Corp., NY, USA) [20] for statistical analysis. During the examination of the study data, the researcher used descriptive statistical methods to identify and sort the ticks. In addition, Microsoft Excel and PowerPoint (2023, Microsoft Office, Microsoft, Washington, USA) were used to construct tables and graphs to express the statistical relationships found for each of the four independent variables (maximum temperature, mean temperature, percentage relative humidity, and rainfall).

### Data validation

A senior entomologist verified the tick species identification made by field technicians by cross-checking a random sample. The validation process reduced the occurrence of errors in tick infestation and species identification.

## Results

**Figure-2:**
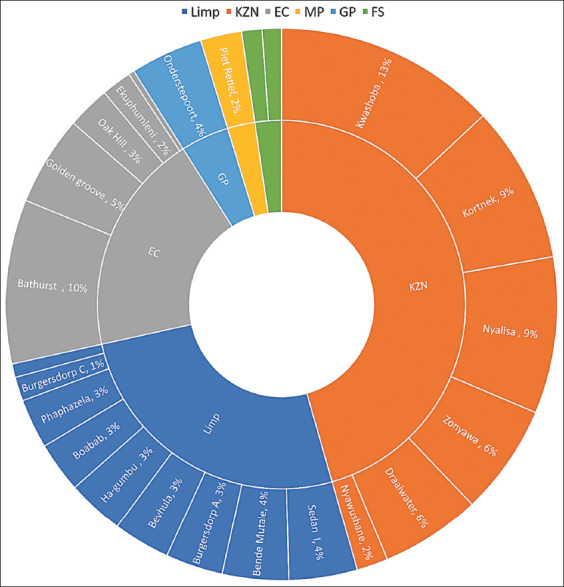
Frequency of ticks per study site.

The distribution of tick infestations across the different geographic regions was categorized by provincial location. KZN (Including Kwashoba, Zonyawa, Nyalisa, Kortnek, Draaiwater, and Nyawushane) exhibited the highest tick infestation, accounting for 46% of the total tick infestation. Subsequently, LP, which encompasses the Tzaneen, Mutase, Musica, and Thohoyandou regions, accounted for 26% of the tick population, as shown in Figure-2. The EC, including East London and Genera, contributed 19% to the overall collection population. GP (Pretoria) recorded 4% of the total count. Finally, the findings in MP (Mkhondo) and the FS (with Bloemfontein as its focal point) each represented 2% of the collected population, as visually represented in Figure-1. Remarkably, Bloemfontein in the FS Province had the lowest number of ticks collected among the various study locations investigated. Figure-1 details the presence of each tick species per study location. In general, the Pongola region in KZN province was found to have the highest diversity of tick species collected for the present study, closely followed by the Tzaneen region in LP province.

### Species frequency

*A. hebraeum* (55.1%) and *R. evertsi evertsi* (13.9%) emerged as the predominant tick species in all study sites, as highlighted in Figure-2 below. However, the infestation rate of several other tick species remained notably low. The less prevalent species included *Harpalus rufipes* (0.8%), *Haemaphysalis silacea* (0.5%), *R. exophthalmos* (0.2%), *Rhipicephalus glabroscutatus* (0.2%), *R. sanguineus* (0.2%), *Ixodes pilosus* (0.1%), and *Rhipicephalus simus* (0.1%). Specific tick species exhibited highly localized distribution patterns. For instance, *I. pilosus* was exclusively found in Gqeberha (Ekuphumleni), *R. glabroscutatus* was confined to the Pongola region, and *R. simus* was solely identified in East London, all in the EC areas. Such findings emphasize the distinct ecological niches occupied by various tick species within the study sites.

### Role of climate in tick infestation

**Figure-3:**
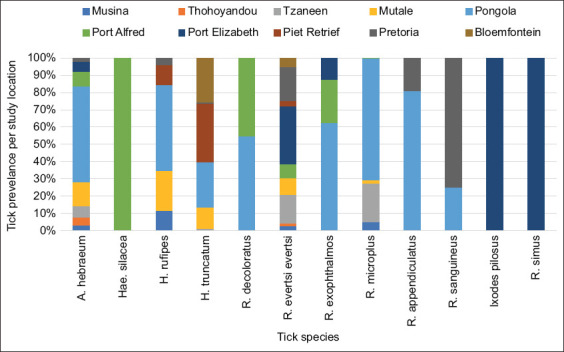
Distribution of total tick infestation per species at different study locations.

**Figure-4:**
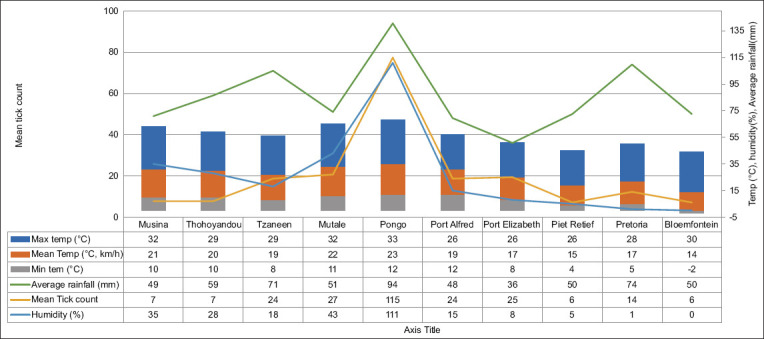
Correlation between seasonal rainfall, average temperature, and average tick infestation across different study locations. The visualization involved aids in understanding the impact of climatic factors on tick infestations, revealing patterns and trends in tick abundance influenced by environmental conditions.

**Figure-5:**
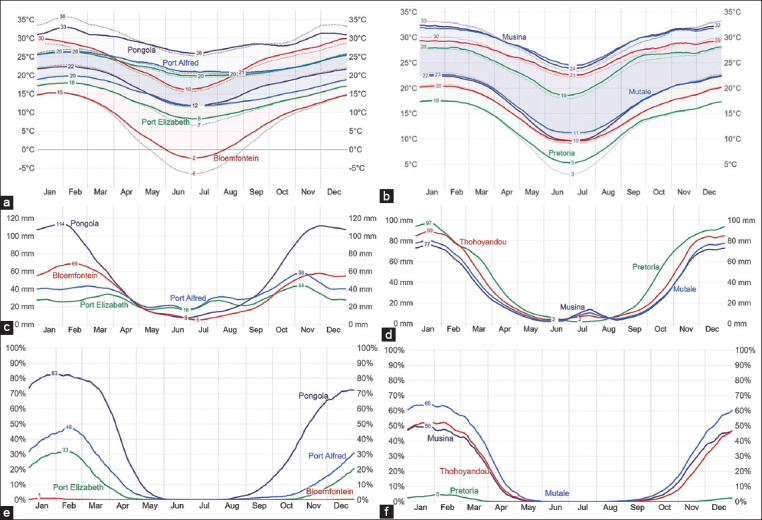
Weather patterns during the tick collection season. (a and b) Show temperature trends in regions including Pongola, Port Alfred, Port Elizabeth, Bloemfontein, Thohoyandou, Mutale, Musina, and Pretoria. (c and d) Depict rainfall distribution in the same regions. (e and f) Present humidity levels across these regions. [Source: Adopted from © WeatherSpark.com].

The distribution patterns for the average infestations of ticks, temperature, humidity, and rainfall during the collection from September 2020 to December 2022 are shown in Figures-4 and 5. Notably, reduced tick collection occurred in three specific regions, namely, Bloemfontein (n *=* 85), Pretoria (n *=* 167), and Mkhondo (n *=* 79), where tick infestation was comparatively subdued. In addition, Figure-3 shows a correlation between tick frequency and average temperature. In regions where mean temperatures rarely exceeded 5°C, tick infestations remained notably low. The prominent mean temperature for Pongola was 23°C, as shown in Figure-3. The region exhibited a high tick infestation, correlated with the highest average rainfall and mean temperature recorded, which were conducive to tick infestation. In addition, the humidity levels in this area were relatively elevated compared to those in other regions investigated. Therefore, the observed temperature and rainfall patterns in the present study were found to influence the variation in tick infestation, thus underpinning the pivotal role played by climatic conditions in the agrogeographic distribution of ticks during the collection time specified.

The climatic conditions during the collection period are shown in Figure-5. In Pongola, high temperatures increased by 3°C (28°C–31°C), with a concurrent rise of 5°C in low temperatures (15°C–20°C). Tzaneen experienced approximately 27°C in terms of high temperatures, accompanied by a 6°C increase in low temperatures (12°C–18°C). Mutale experienced a 3°C rise in high temperatures (28°C–31°C), along with a simultaneous surge of 6°C in low temperatures (15°C–21°C). Gqeberha recorded a 4°C increase in high temperatures (20°C–24°C) and a 5°C rise in low temperatures (11°C–15°C). The high temperatures of Mkhondo increased by 2°C (22°C–25°C), while the low temperatures of the town increased by 6°C (7°C–13°C). Pretoria underwent a 3°C increase in high temperatures (24°C–27°C) and a 5°C increase in low temperatures (11°C–16°C). Thohoyandou experienced a 3°C increase in high temperatures (26°C–29°C) and a simultaneous 6°C increase in low temperatures (13°C–19°C). Bloemfontein experienced a 6°C rise in high temperatures (22°C–28°C) and a notable increase of 10°C in low temperatures (3°C–13°C). Finally, in Bathurst, high temperatures increased by 3°C (21°C–24°C), with a 4°C rise in low temperatures (13°C–17°C).

## Discussion

This study examined three crucial factors, such as average rainfall patterns, tick frequency, and temperature range in predicting Ixodid tick distribution in South African cattle across different agroclimatic zones. Across the sampled regions, a spectrum of tick species was identified, including *A. hebraeum*, *Rhipicephalus* (*Boophilus*) *decoloratus*, *R. evertsi evertsi*, *H. silacea*, *H. rufipes*, *Hyalomma marginatum*, *I. pilosus*, *R. exophthalmos*, *R. glabroscutatus*, *R*. (*Boophilus*) *microplus*, *R. appendiculatus*, *R. simus*, and *R. sanguineus*, were identified. The present study underscores temperature’s significant impact on Ixodid tick survival and infestation. The significance of temperature in tick populations is emphasized by these discoveries. According to Eisen *et al*. [21], the impact of temperature on the survival and distribution of Ixodid tick species in the eastern and western parts of North America over the past two decades has been significant. The study sites, favorable for tick infestation during spring and summer, exhibited suitable temperature profiles.

Interestingly, areas characterized by relatively low temperatures during the collection season (summer), such as Pretoria (n = 139), Mkhondo (n = 79), and Bloemfontein (n = 76) recorded relatively low tick infestations. This observation aligns with the research conducted by Ogden *et al*. [12], which suggests that exophilic ticks, which reside outside their hosts, exhibit reduced host-biting tendencies under extremely low temperature conditions. The current observations highlight the complex relationships among climate, tick behavior, and distribution patterns. This underscores the multifaceted nature of tick-related research in agroclimatic contexts.

### Effects of climate change on *Amblyomma* species

*A. hebraeum*, classified as a tick species [22], was found to exhibit a widespread presence at the study sites. The making of such a discovery agrees with the findings reported by Mapholi *et al*. [23], who identified the species as being the most prevalent throughout the collection period. This species exhibits its highest frequency from October to January. The existence of livestock farming with cattle, goats, and sheep in the explored regions facilitated the emergence and spread of tick species *A. hebraeum*.

Animal movement significantly contributes to the occurrence of *A. hebraeum* outbreaks, according to Mandara [24]. The presence of *Amblyomma* species in new locations is likely due to both local movement of wild animals and long-distance translocation of livestock. The Pongola region, with its game reserves and cattle herds sharing water sources, recorded substantial numbers of this tick species. In LP’s Mutale region, close to Kruger National Park, there was a significant count of *A. hebraeum*. Horak *et al*. [5] suggest that warthogs, eland, kudu, giraffes, buffalo, and rhinoceros are the essential hosts for this tick species, possibly explaining its prevalence in cattle grazing near game reserves.

Seasonal trends in *A. hebraeum* activity align with observations made by Panadero *et al*. [25] and Mapholi *et al*. [23], indicating that the maximum population of *A. hebraeum* on cattle in LP tends to occur between October and February. In the present study, a similar pattern was observed, with *A. hebraeum* representing 55.1% of the overall tick collection population, which prevailed across all farms examined in LP. Notably, Pongola (KZN) emerged as a hotspot for *A. hebraeum* infestation, with a remarkable 95% of identified ticks (n *=* 1023/3, 435) being recorded in this region. This abundance can be attributed to the peak of the tick when the ticks are most active feeding on hosts during early summer, as reported by Horak and Spickett [26]. The observed behavior aligns with the conclusions of Schroder *et al*. [27], who reported that *A. hebraeum* emerged as the predominant tick species across several regions in South Africa, notably in LP and KZN, reaching its highest prevalence during the early summer months. Furthermore, the species exhibits a preference for moderate temperatures, with increased sensitivity in the range of 26°C–29°C. However, elevated temperatures above 35°C might result in decreased populations of *A*. *hebraeum* ticks in various locations, as indicated by Tagwireyi *et al*. [28]. The Pongola (KZN) and Mutale (LP) regions, both experiencing annual mean temperatures of 20°C and 25°C, respectively, recorded the highest infestations of this species on cattle.

#### Effects of climate change on Rhipicephalus species

*R. evertsi evertsi* emerged as the second most prevalent tick species in the present study, accounting for 13.9% of the total tick population. This species exhibited a wide distribution, being present at all collection sites. Schroder and Reilly [29] indicated that *R. evertsi*, characterized as a two-host tick, thrives in humid and warm climates. Its adaptability can persist in various biomes, including deserts, steppe regions, savannas, and temperate climates. Moreover, Mapholi *et al*. [23] affirmed that *R. evertsi* is the most extensively distributed species among all *Rhipicephalus* species found in Africa. The wide distribution can be attributed to its ability to thrive in diverse ecological conditions. In addition, *R. evertsi* plays a role in the transmission of anaplasmosis, which is a significant cattle disease. Furthermore, these tick species possess salivary toxins that can induce paralysis in calves, as documented by Makwarela *et al*. [30].

*R. decoloratus*, comprising 11.9% of the total tick population identified, emerged as the third most prevalent tick species in the present study. The species was collected primarily in the EC and KZN regions of South Africa. These findings align with the research conducted by Spickett Arthur [31], who reported that *R*. (*Boophilus*) *decoloratus* was commonly found in the coastal and adjacent regions of the KZN and EC provinces, as well as in the three northern provinces of South Africa (GP, MP, and LP). The present study did not detect the presence of *R*. (*Boophilus*) *decoloratus* in LP and GP, contrary to the findings made in previous studies by Schroder *et al*. [27] and Spickett Arthur [31], which were conducted around the time of the same peak seasons. The variation involved could be attributed to the gradual replacement of *R*. (*Boophilus*) *decoloratus* by the Asian species *R*. (*Boophilus*) *microplus*, as suggested by Nyangiwe *et al*. [32]. In some regions, such as Mozambique, *R*. (*Boophilus*) *microplus* has completely displaced *R*. (*Boophilus*) *decoloratus*, as documented by Horak *et al*. [22]. Moreover, *R*. (*Boophilus*) *decoloratus* prefers warm and humid environments.

The findings of the present study are consistent with those of a study conducted in warm and humid regions of Ethiopia [33], where *R*. (*Boophilus*) *decoloratus* was recovered from coastal areas. Coastal regions are characterized by high humidity, which is facilitated by winds blowing from the sea to the land. Notably, in Bathurst, where *R*. (*Boophilus*) *decoloratus* was identified; the climate tends to feature short, warm, and humid summers, along with a relatively wet season, lasting 6.5 months, from October to April, creating a favorable habitat for *R*. (*Boophilus*) *decoloratus*. Similarly, the Pongola region, which is marked by hot and humid winters and warm summers, maintains clear skies throughout the year, contributing to tick infestations. According to this study, *R*. (*Boophilus*) *decoloratus* remains common in areas of high humidity such as Genera, EC. However, the potential displacement of these species warrants further investigation.

According to Kumar *et al*. [34], *R*. (*Boophilus*) *microplus* is the primary tick involved in the global spread of parasitic diseases affecting cattle, the tick being linked to economic losses in cattle. In the present study, the presence of *R*. (*Boophilus*) *microplus* was confirmed in both the LP and KZN regions. The results obtained agree with the conclusions drawn by Horak *et al*. [22] that most of the collections of this species were conducted in the savanna biome of northern LP and the grass biome of the southeast KZN Province. The habitats of *R*. (*Boophilus*) *decoloratus* and *R*. (*Boophilus*) *microplus* have similar climatic characteristics. Both species thrive in environments with ideal rainfall of approximately 750 mm and temperatures ranging between 18°C and 20°C. Although *R*. (*Boophilus*) *microplus* is more widely distributed throughout South Africa; both species can co-exist in a few locations. In the present study, the co-existence relationship between the two species was observed on some farms in KZN and EC provinces. However, interspecific competition between the two species still requires a full assessment. The present findings imply that it is unlikely for *R*. (*Boophilus*) *microplus* to survive in most regions where it is cold if it spreads to new habitats, such as through the movement of cattle. Due to South Africa’s recurring tropical climate conditions, the *R*. (*Boophilus*) *microplus* species is found throughout the country according to Canevari *et al*. [35]. Typically, temperatures ranging from 20°C to 35°C and relative humidity exceeding 70% are considered optimal for the survival of engorged females and eggs. The seasonality of the climate, according to Marques *et al*. [36], is an important component in the lifecycle of *R*. (*Boophilus*) *microplus*, with variations in this aspect affecting the number of generations concerned, both serving to grow the population and enabling dispersal.

In the present study, the presence of *R*. (*Boophilus*) *microplus* was confirmed in the LP and KZN regions of South Africa. These findings align with the conclusions drawn by Horak *et al*. [22], who reported that most collections for this species occurred in the savanna biome of northern LP and the grass biome of southeast KZN.

*R. appendiculatus* demonstrates a propensity to avoid arid regions, particularly those that receive little or no rainfall during the late winter and early spring seasons, as noted by Lynen *et al*. [37]. These tick species thrive in areas characterized by moderate mean temperatures during the same period. In the present study, *R. appendiculatus* was identified in the Pongola region, where the mean annual rainfall averages approximately 806 mm and the altitude is approximately 234 m above sea level. This study observed a prevalent infestation of *R. appendiculatus* ticks in the ears of cattle, with fewer ticks on their backs. This observation supports the findings of Mapholi *et al*. [23], who also reported a higher infestation of *R*. *appendiculatus* in the ears.

The frequency of the brown ear tick *R. appendiculatus* is notably high in Highveld areas situated above 600 m above sea level. The areas concerned are characterized by substantial rainfall (of at least 650 mm/year), relatively low temperatures ranging from 10°C to 30°C, and sufficient vegetation [38]. The temperature in Pongola typically fluctuates between 12°C and 33°C, with rare instances of temperatures falling below 8°C or exceeding 36°C annually. Such temperature conditions tend to align with the findings reported by Madder *et al*. [39], which suggest that *R. appendiculatus* adults can often survive for up to 2 years at temperatures of 22°C and 87% relative humidity. Furthermore, *R. appendiculatus* serves as the primary vector for one of the most significant bovine parasite diseases in East and Central Africa, caused by the protozoan *Theileria parva*, commonly referred to as east coast fever, as reported by Olds, Mason [40].

### Effects of climate change on *Hyalomma* species

In the present study, three species of the *Hyalomma* genus, namely, *H. marginatum*, *H. rufipes*, and *H. truncatum*, were identified. Among these species, *H. marginatum* was the most dominant and was found to coexist with *H. truncatum* in the FS region. Notably, *H. marginatum* was found to be particularly dominant in KZN, with several ticks being located (n *=* 56/164). Duscher *et al*. [41] suggested that warm temperatures during autumn can lead to the molting of nymph populations into adults, resulting in decreased winter mortality, as adult ticks are generally more resistant to cold temperatures than nymphs. The warm temperatures in the Pongola region, KZN appeared to favor the survival and frequency of *H. marginatum*, which was found in abundance in that region. This is supported by Estrada-Peña *et al*. [42], who demonstrated the impact of temperature and water vapor on tick survival and development and the potential spread of tick populations under different climate scenarios. The findings of the present study also align with the results reported by Estrada-Peña *et al*. [43], who indicated that high densities of *H. marginatum* were associated with spring, characterized by a mean temperature of 27°C and a relative humidity of 84%. Furthermore, the results obtained validated the observations made by Horak *et al*. [22] that this species tends to be found in regions with moderately dry environments. The development of tick populations is suggested to depend on temperatures during spring and summer (starting in September and December), as unfed adults can survive through the next season if temperatures are sufficiently high to allow molting before the harsh winters set in. In contrast, *H. rufipes* and *H. truncatum* were found in fewer numbers in the regions of LP, GP, MP, and the FS provinces. This is consistent with the findings of Bryson *et al*. [44], who reported low infestations of H. *rufipes* in MP.

### Effects of climate change on *Ixodes* and *Haemaphysalis* species

*I. pilosus*, a tick species with specific habitat preferences, prefers microclimatic conditions with a relative humidity of at least 80% to prevent desiccation during its prolonged non-parasitic stages. They are typically found in regions with moderate to high rainfall and good vegetation cover, as reported by Gray [45]. Climate change is expected to influence the survival, peak activities, and distribution of *I. pilosus*. This impact is especially significant in regions with low summer precipitation and high summer temperatures, where the risk of desiccation can affect their numbers and disease spread, as noted by Gilbert *et al*. [46].

In the present study, *I. pilosus* was flavored by the temperatures in Genera, EC. The findings of the present study align with those of Yawa *et al*. [47], who reported the presence of *I. pilosus* in the EC, where annual rainfall averages 624 mm and temperatures range between 13°C and 29°C in summer and between 1°C and 12°C in winter. The environmental conditions in Gqeberha and the EC suit *I. pilosus*, highlighting the importance of climate and habitat factors in the distribution of these tick species. This preference for high humidity is consistent with the findings of studies on *Ixodes ricinus*, a related tick species, which also shows a dependence on microclimate for survival and distribution [48, 49].

Limited information exists on the ecological and host interactions of *H. silacea* in South Africa, particularly in the EC Province. Londt and Whitehead [50] and Norval [51] both provided valuable insights into the distribution and activity patterns of this tick species, with Norval [51] highlighting its presence in the EC and its preference for artiodactyl hosts, and Londt and Whitehead [50] discussing its activity peaks during the winter months. Horak *et al*. [52] further contributed to our understanding by confirming the presence of *H. silacea* in the FS Province and its prevalence on cattle in the EC Province. However, more research is needed to fully understand the ecological and host interactions of these tick species in South Africa.

The results of the present study align with the observations documented by Londt and Whitehead [50], indicating that *H. silacea* tends to inhabit environments distinguished by a dense canopy of bushes or trees and limited herbaceous leaf litter, especially in coastal areas.

Despite minimally impacting tick infestation during collection, the study’s data on humidity conditions provide insight into humidity’s significance in the entire lifecycle of Ixodid ticks. The present study reveals that temperature significantly influences immediate tick infestation, acting as the primary determinant of tick infestation patterns. In regions with temperatures exceeding 18°C, cattle are more prone to high tick infestation due to the favorable environment for tick acquisition.

## Conclusion

This study sheds light on the association between tick infestation and climate factors, specifically temperature and humidity. The findings serve as a foundation for comprehending tick distribution and tick eradication measures. The complex impacts of climate change extend to ecosystems, host populations, and the lifecycles of ticks and tick-borne disease-causing pathogens. The study’s scope was regional and limited to certain seasons. To fully understand tick distribution patterns and disease dynamics, more research is needed on the impact of the season and other factors. Effective strategies for managing tick and tick-borne diseases depend on in-depth comprehension of climate change’s influence on tick ecology and disease transmission, particularly in regions where these diseases pose a major risk to livestock and human health.

## Data Availability

The data that underpin the study’s conclusions are available from the authors, but there are limits on their use and they are not made available to the public. However, with permission from the University of South Africa and on reasonable request, data will be made available by the authors.

## Authors’ Contributions

TGM and NOM: Conceptualized the study. TGM: Conducted the experiments, laboratory works, and data collection. TGM and NN: Sample collection. TGM: Performed data analysis. TMS, AD, LTN, and NOM: Supervised the study and revised the manuscript. TGM: Wrote the original draft. All authors have read, reviewed, and approved the final manuscript.
